# 기혼여성의 가족가치관, 생식건강지식이 생식건강증진행위에 미치는 영향

**DOI:** 10.4069/kjwhn.2022.11.28.1

**Published:** 2022-12-29

**Authors:** Sun Jeong Yun, Hye Young Kim

**Affiliations:** College of Nursing Science, Keimyung University, Daegu, Korea; 계명대학교 간호대학

**Keywords:** Health promotion, Knowledge, Reproductive health, Values, 건강증진, 지식, 생식건강, 가치

## Abstract

**Purpose:**

Based on the World Health Organization framework on reproductive health, this descriptive correlational study investigated the factors affecting reproductive health-promoting behaviors of married women, with a focus on family values and reproductive health knowledge.

**Methods:**

A cross-sectional survey was conducted on 170 married women between the ages of 25 and 49 years living in Daegu, Korea. The general and reproductive health characteristics, family values, and reproductive health knowledge of married women were identified, as well as factors affecting reproductive health-promoting behaviors. A questionnaire survey was administered to investigate the impact of various factors on reproductive health-promoting behaviors.

**Results:**

Positive correlations were shown for health promotion behaviors with family values (r=.78, *p*<.001) and reproductive health knowledge (r=.55, *p*<.001). Family values (β=.35, *p*<.001) and reproductive health knowledge (β=.24, *p*<.001) were identified as factors influencing reproductive health-promoting behaviors. According to the regression model, the explanatory power of factors affecting reproductive health-promoting behaviors among married women was 51.2%.

**Conclusion:**

A history of reproductive diseases, family values, and reproductive health knowledge were identified as factors influencing reproductive health-promoting behaviors. These results provide basic data for the development of a reproductive health-promoting program, including a positive approach to reproductive health among married women, and can serve as a basis for further research on intervention strategies.

## Introduction

국내 고용, 소득, 가족 및 사회정책 등의 변화는 결혼 행태에 영향을 줌으로 혼인 종류의 다양화, 초혼 연령 증가, 출산 연령 증가로 이어지고 이로 인해 여성 생식건강 요구가 증가되었다[1]. 국내에서는 2009년 모자보건법 개정을 통하여 생식건강이라는 용어가 새로 등장하였다[2]. 생식건강의 의미는 임신, 분만, 산욕기 기간 이외에도 여성의 전 생애에 걸쳐 생식기 계통과 그 기능 및 변화 과정에서 단순히 질병이 없는 상태뿐만 아니라 육체적·정신적·사회적으로 안녕한 상태이다[3]. 모자보건법 개정과 함께 우리나라에서는 모자보건사업으로 여성 인구의 생식건강, 생식권과 난임부부 지원, 안전한 임신 및 출산 보장, 임신 소모의 최소화 등 가임기 여성을 대상으로 생식건강 증진을 지원하고 있다[1]. 또한 국가와 지방자치단체는 모자보건사업의 확대 차원에서 체계적 실태조사 및 교육 등을 제공하고 인구 변화에 따른 접근을 시도하며 생식건강을 관리하는 사업을 지속적으로 개발·운영 및 평가하고 있다[1,4].

최근 우리나라 통계청[5] 자료에 따르면 초혼 연령이 2011년 여성 평균 연령 29.1세와 비교하여 2021년에는 31.1세로 높아졌고 연령별 혼인율(해당 연령 인구 1천 명당 혼인 건수)은 2021년에 들어서 30대 초반 혼인율이 20대 후반 혼인율보다 높게 나타났다. 그리고 출산율은 경제협력개발기구(Organisation for Economic Co-operation and Development, OECD) 국가 중에서 가장 빠르게 하락하여 2018년 합계 출산율이 OECD 국가 중 유일하게 1명 이하로 떨어졌고, 2021년에는 0.81명으로 OECD 최하위를 기록하였다[6]. 이러한 현상을 바탕으로 여성 생식건강과 가족가치관을 살펴볼 필요가 있다. 생식건강은 여성이 출산능력을 가지고 출산 여부, 출산 시기 및 출산 횟수를 자유롭게 선택할 수 있는 권리[7]를 포함하여 인구 및 가족 계획 사업으로 인식되어 개인, 가족, 지역 사회, 국가의 사회경제 발전을 기반으로 한 평생 건강의 핵심 요소로 본다[1]. 그리고 가족가치관은 자녀에 대한 중요성 및 부모됨에 대해 개인이 가지는 가치관이며 결혼, 출산, 자녀, 양육 등에 대한 주관적 인식에 가장 큰 영향을 주는 광의적 개념으로 정의된다[8]. 가족가치관의 자녀 가치관은 부모가 자녀를 갖고 양육하려는 동기로 이해되고 자녀 출산 동기와 출산 계획에 영향을 미친다고 한다[9]. 이처럼 여성 생식건강과 가족가치관은 밀접한 관계가 있다. 특히 기혼여성의 생식건강과 가족가치관은 여성 개인뿐만 아니라 가족의 건강과도 직결되고, 건강한 임신과 출산의 결과는 미혼 여성들에게도 광범위한 영향을 미치는 만큼 기혼여성의 생식건강에 관심을 가지는 것은 중요하다[10]. 그러나 기혼여성을 대상으로 가족가치관과 개인의 생식건강증진행위를 함께 고찰한 연구는 부족한 실정이다. 이에 본 연구자는 가족의 형태와 기능, 가치관의 변화에 따른 기혼여성의 가족가치관을 확인하고 여성의 결혼, 출산 행태를 고려한 생식건강증진행위 중재 개발에 실천적 전략을 위한 기초자료를 제공하고자 한다.

생식건강지식은 생식기계 질병 및 장애, 임신 및 출산, 가족 계획, 인공 임신중절, 성병, 성 건강 등의 생식건강 문제[11]에 대한 지식을 의미하며 신체적, 생리적 현상 중심인 성 지식과는 차이가 있다. 최근 자유로운 성문화에 노출이 쉬워졌고, 평균 성행위의 연령이 낮아지면서 안전하고 올바른 생식건강증진행위를 위한 생식건강지식의 정도를 확인하는 것이 더욱 중요해지고 있다[12]. 생식건강에 대한 올바른 이해와 정확한 지식은 생식건강행위 실천율을 높이고, 성 문제를 예방하며, 책임감 있는 출산에 대한 의사결정을 할 수 있게 돕는 중요한 요소가 된다[13]. 또한 국가와 지방자치단체의 여성생식건강에 대한 사회적 지지체계를 구축하고 방안을 모색하는 상황에서 생식건강지식의 정도를 확인해 볼 필요가 있다.

생식건강증진행위는 안전 성행위, 성행위에 대한 책임감, 생식기 질환의 조기 발견을 위한 생식기 건강 관리, 성병 예방, 가족 계획, 임신 및 출산, 생식기 위생 관리 등, 생식건강에 긍정적 행위를 포함한다[13]. 그리고 Jo 등[12]의 연구에서는 생식건강증진행위를 안전한 성행위, 생식기 관리, 불임 관련 건강 관리, 성적인 책임을 포함한 심리사회적 생식건강으로 식별하며 체계적으로 분류하였다. 생식건강증진행위에 대한 선행 연구를 살펴보면, 여성 결혼이민자의 생식건강을 증진하기 위한 연구[13], 난임 유무에 따른 여성의 생식건강증진행위를 비교한 연구[14], 가임기 염증성 장질환 여성을 대상으로 한 연구[15], 미혼 대학생들을 대상으로 한 연구[9,12]가 많았다. 이는 생식건강증진행위가 여성 개인의 성 건강에 대한 책임감과 안전한 성 태도에 영향을 줄 수 있기 때문[16]으로 여겨진다. 그러나 본 연구자는 기존의 연구와 달리 생식건강이 개인뿐만 아니라 배우자 및 자녀와 직·간접적으로 관련되어 있고, 인공 임신중절 경험률이 높고, 생식기 건강문제를 가지고 있는 빈도가 높은[13] 기혼여성들을 대상으로 생식건강증진행위 연구가 필요하다는 것을 확인하였다.

세계보건기구(World Health Organization)와 국제연합(United Nations) 총회에서는 생식건강의 의료 분야 연구 및 정책 개발과 생식건강 서비스의 보편적 접근을 보장하기 위한 구체적 목표를 설정하였고, 국내 국민건강증진종합계획에서는 인구 정책의 맥락으로 저출산 현상에 대한 가족과 생식건강에 대해 사회적인 관심이 증가하였다[17]. 이에 본 연구자는 생식건강의 통합적 관점에서의 연구 필요성을 고려하여 기혼여성을 대상으로 가족가치관, 생식건강지식이 생식건강증진행위에 미치는 영향을 확인하여, 기혼여성들의 생식건강에 긍정적 접근을 위한 생식건강증진행위 프로그램 개발에 기초자료를 제공하고, 다각적인 시각으로 중재 전략의 근거가 되는 연구 자료를 제공하고자 한다.

본 연구의 목적은 기혼여성의 가족가치관, 생식건강지식이 생식건강증진행위에 미치는 영향을 파악하고자 하는 것이며, 구체적인 목적은 다음과 같다.

1) 기혼여성의 가족가치관, 생식건강지식 및 생식건강증진행위 정도를 확인한다.

2) 기혼여성의 일반적 및 생식건강 특성에 따른 생식건강증진행위 정도의 차이를 확인한다.

3) 기혼여성의 가족가치관, 생식건강지식 및 생식건강증진행위 간의 상관관계를 확인한다.

4) 기혼여성의 생식건강증진행위에 미치는 영향요인을 확인한다.

## Methods

Ethics statement: This study was approved by the Institutional Review Board of Keimyung University (No. 40525-202203-HR-005-02). Obtaining written informed consent was exempted because the survey was completely anonymous and considering the online survey design.

### 연구 설계

본 연구는 기혼여성의 가족가치관, 생식건강지식이 생식건강증진행위에 미치는 영향을 파악하기 위한 상관성 조사 연구설계로, STROBE (https://www.strobe-statement.org/) 지침에 근거하여 기술하였다.

### 연구 대상 및 표집 방법

본 연구 대상자는 25세 이상 49세 미만의 기혼여성으로 본 연구의 목적과 취지를 이해하고 자료 수집에 동의한 대상자를 편의표집하였다. 제외 기준은 현재 생식기 질병을 진단받고 치료 중인 자, 지남력 장애 및 정신과 질환을 진단받고 치료 중인 자로 선정하였다. 현재 생식기 질병을 진단받고 치료 중인 자는 건강상태를 지각하고 건강 회복을 위한 긍정적 행위를 실천[18]하므로 연구 결과에 영향을 줄 수 있다고 판단하여 제외하였다. 연구 수행에 필요한 표본 크기는 Lee [19]의 선행 연구를 바탕으로 G*Power 3.1.9.2 프로그램을 이용하고, 다중회귀분석에서 임의 추정 예측변수 15개, 효과크기 .15, 유의수준 .05, 검정력 .80으로 총 139명으로 산출하였으나, 탈락률 20%를 고려하여 177명을 모집하였다. 설문지 중 응답이 불완전하거나 불성실한 2부를 제외한 170부를 최종 자료 분석에 활용하였다.

### 연구 도구

본 연구에서 사용된 주요 변수를 측정하기 위한 구조화된 도구는 이메일을 통하여 개발자에게 도구 사용에 대한 승인을 받았다.

#### 생식건강증진행위(reproductive health-promoting behaviors)

생식건강증진행위는 Jo 등[12]이 대학생을 대상으로 개발하고 Lee와 Lee [14]가 기혼여성에게 사용하기 위하여 수정, 보완한 도구를 사용하였다. 생식건강증진행위 측정도구는 안전 성행위 4문항, 성행위 책임감 4문항, 생식기 건강 관리 4문항, 성병 예방 3문항, 생식기 위생 관리 3문항 등 총 18문항으로 구성되어 있다. 각 문항은 5점 Likert 척도로 ‘전혀 그렇지 않다’ 1점, ‘아주 그렇다’ 5점으로 점수를 구하며 점수가 높을수록 생식건강증진행위 수행도가 높음을 의미한다. 개발 당시 도구의 신뢰도는 Cronbach’s α=.88, Lee와 Lee [14]의 연구에서 신뢰도는 Cronbach’s α=.76–.90이었으며 본 연구에서는 Cronbach’s α=.81이었다.

#### 가족가치관(family values)

가족가치관은 Seo [20]가 선행논문을 재구성하여 수정, 보완한 측정도구를 사용하였다. 자녀가치와 부모됨가치로 구분되며, 자녀가치는 자녀의 필요성 및 중요성에 관한 3문항, 부모됨가치는 부모가 되고, 부모로서 자녀를 양육하는 것에 대한 중요성에 관한 4문항으로 총 7문항으로 구성되어 있다. 7점 Likert 척도로 ‘전혀 그렇지 않다’ 1점에서 ‘매우 그렇다’ 7점까지의 범위를 갖는다. 점수가 높을수록 전통적인 가족가치관이 강한 것을 의미한다. Seo [20]의 연구에서 자녀가치 신뢰도는 Cronbach’s α=.77, 부모됨가치 신뢰도는 Cronbach’s α=.84로 나타났다. 본 연구에서는 자녀가치 신뢰도는 Cronbach’s α=.79, 부모됨가치 신뢰도는 Cronbach’s α=.80이었다.

#### 생식건강지식(reproductive health knowledge)

생식건강지식은 Park과 Choi [13]가 개발한 생식건강지식 척도를 Cho [21]가 수정, 보완한 측정도구를 사용하였다. 문항은 생식기의 구조 및 기능 6문항, 임신 및 출산 11문항, 피임 및 성 매개 감염 12문항, 생식기 암 5문항 등 총 34문항으로 구성되어 있다. 정답을 체크하면 1점, 오답 및 ‘모르겠다’로 체크하는 경우는 0점을 부여하고 점수의 범위는 최저 0점에서 최고 34점까지이며(가능점수 범위: 0-34점), 점수가 높을수록 생식건강지식이 높음을 의미한다. 개발 당시 도구의 신뢰도는 Kuder-Richardson 20 (KR-20) .79였으며, Cho [21]의 연구에서 신뢰도는 KR-20 .88이었다. 본 연구에서는 KR-20 .82이었다.

#### 대상자 특성

본 연구는 도구의 하위영역별 비교 확인을 위하여 결과는 평균평점으로 제시하였다. 선행 연구를 바탕으로 연령, 종교, 교육수준, 직업, 소득수준의 일반적 특성과 결혼 기간, 자녀 유무, 생식건강 상태, 피임 방법, 과거 생식기 질병 경험, 생식건강 교육 여부, 생식건강 교육 필요성의 생식건강 관련 특성으로 총 12문항을 구성하였다.

### 자료 수집

자료 수집은 2022년 5월 18일부터 2022년 7월 2일까지 진행되었다. 대구광역시 소재지 초등학교 1곳, 의료기관 2곳, 직장인 전용 익명 소셜 네트워크 서비스(social networking service)에서 담당자의 동의를 얻은 후 대상자 모집 공고문을 공지하였다. 그리고 본 연구의 필요성 및 목적을 이해하고 대상자 기준에 부합한 자는 온라인 설문을 위하여 QR (quick response) code, URL (uniform resource locator) 단축 서비스를 활용하여 온라인 접속을 하고, 개인정보 수집 및 이용 동의 항목에 동의 한 대상자에 한하여 온라인 설문이 진행되도록 프로그램을 설계하였다. 설문지 작성에는 약 15분 정도의 시간이 소요되었으며, 설문 완료 후 연구 보상을 위하여 모바일로 커피 쿠폰을 제공하였다.

### 자료 분석 방법

수집된 자료는 IBM SPSS ver. 22.0 (IBM Corp., Armonk, NY, USA) 통계 프로그램을 이용하여 분석하였으며 구체적인 방법은 다음과 같다.

1) 대상자의 일반적 및 생식건강 특성은 실수와 백분율, 평균과 표준편차로 분석하였다.

2) 대상자의 가족가치관, 생식건강지식, 생식건강증진행위는 평균, 표준편차로 분석하였다.

3) 대상자의 일반적 및 생식건강 특성에 따른 생식건강증진행위 차이는 independent t-test, 일원분산분석으로 분석하고, 사후 검정은 Scheffé test를 이용하였다.

4) 가족가치관, 생식건강지식, 생식건강증진행위의 상관관계를 파악하기 위하여 Pearson correlation으로 분석하였다.

5) 대상자의 생식건강증진행위에 미치는 영향 요인을 파악하기 위하여 위계적 회귀분석(hierarchical analysis)으로 검증하였다.

## Results

### 대상자의 일반적 및 생식건강 관련 특성

대상자의 연령은 25세 이상 49세 미만(평균 32.28±0.31세), 종교는 ‘있음’이 77.6%, 교육수준은 4년제 대학 졸업이 72.4%, 월 수입평균은 251.09±0.58만원으로 나타났다. 생식건강 특성에서 결혼 기간은 1–2년 사이가 32.9%, 자녀는 ‘있음’이 71.8%, 현재 생식건강 상태에 대하여 ‘보통’이 59.4%, 피임 방법은 콘돔이 54.1%, 과거 생식기 질병 경험은 월경 장애가 40.0%, 생식건강 교육 경험은 ‘없음’이 59.2%, 생식건강 교육 필요성은 ‘예’가 87.6%였다(Table 1).

### 대상자의 생식건강증진행위, 가족가치관 및 생식건강지식

생식건강증진행위는 평균 평점 5점 만점에 3.18점으로, 하부 요인 중 안전 성행위가 3.32점으로 가장 높았고, 생식기 건강 관리가 3.09점, 생식기 위생 관리가 3.09점으로 가장 낮았다. 대상자의 가족가치관은 평균 평점 7점 만점에 4.40점, 생식건강지식은 평균 평점 34점 만점에 27.98점으로 나타났다. 세부 항목으로 부모됨가치는 평균 4.45점, 자녀가치는 평균 4.36점이었다(Table 2).

### 대상자의 일반적 및 생식건강 관련 특성에 따른 생식건강증진행위 차이

대상자의 일반적 및 생식건강 특성에 따른 생식건강증진행위의 차이는 다음과 같다 생식건강증진행위는 대상자 교육수준은 대학교가 평균 3.51점, 고등학교 이하가 평균 3.27점으로 집단 간 유의한 차이가 있었다(F=1.32, *p*=.011). 결혼 기간은 1년 이상, 2년 이하가 평균 3.65점, 3년 이상, 4년 이하가 평균 3.40점으로, 결혼 기간 1년 이상 2년 이하에서 생식건강증진행위가 높게 나타났다(F=0.33 *p*=.011). 피임 방법은 콘돔이 평균 3.56점, 월경 주기가 평균 3.44점으로, 콘돔이 가장 높게 나타났다(F=1.01, *p*=.021). 과거 생식기 질병 경험에서 생식기 감염은 평균 3.67점, 월경 장애가 3.49점으로 생식기 감염 질병 및 증상에 따른 생식건강증진행위 차이가 가장 높게 나타났다(F=0.55, *p*=.018) (Table 1).

### 대상자의 생식건강증진행위, 가족가치관 및 생식건강지식 상관관계

대상자의 가족가치관과 생식건강지식은 상관 관계가 유의하지 않았으나, 생식건강증진행위와 가족가치관(r=.78, *p*<.001), 생식건강지식(r=.55, *p*<.001)은 정적 상관관계로 통계적으로 유의한 결과가 나타났다. 즉, 대상자의 가족가치관과 생식건강지식이 높을수록 대상자의 생식건강증진행위 수행도가 높은 것으로 나타났다(Table 3).

### 대상자의 생식건강증진행위에 미치는 영향요인

대상자의 일반적 및 생식건강 특성이 생식건강행위에 영향을 주므로 1단계에 넣고, 주요변수인 가족가치관과 생식건강지식의 중요성을 확인하고자 2단계에 넣어 위계적 다중회귀분석을 실시한 결과는 다음과 같다(Table 4).

본 연구에서 회귀모형에 대하여 첫째, 잔차의 산포도를 이용하여 종속변수와 독립변수 간의 선형관계와 등분산성을 확인하였다. 둘째, 잔차의 독립성을 확인하기 위하여 Durbin-Watson 지수를 확인한 결과 1.744로 2에 가까워, 자기 상관이 없어 오차항 간 서로 독립적이었다. 셋째, 정규성 검정을 위하여 Shapiro-Wilk test를 통하여 확인한 결과 변수 모두 유의확률 0.05보다 높아 정규분포를 따르는 것을 확인하였다. 넷째, 독립변수 간의 상관관계를 확인하기 위하여 다중공선성을 분산팽창지수(variance inflation factor)를 통하여 확인한 결과, 모두 10 미만으로 다중공선성의 문제가 없는 것으로 확인하였다.

분석 결과 1단계에서는 일반적 및 생식건강 특성 중 생식건강증진행위에 차이가 있었던 변수인 교육수준, 결혼 기간, 피임 방법, 과거 생식기 질병 경험을 투입하였다. 그리고 유의한 차이가 있었던 변수들의 가장 큰 값을 기준으로 교육수준에서 대학교, 결혼 기간 1–2년, 피임 방법 중 콘돔, 과거 생식기 질병 경험에서 월경 장애를 더미변수 처리하여 투입하였을 때, 회귀모형은 유의하였으며(F=18.48, *p*<.001) 설명력은 21.5%로 나타났다. 2단계에서는 가족가치관, 생식건강지식을 투입했을 때 회귀모형은 유의하였으며(F=41.07, *p*<.001), 설명력 51.2%로 나타났다. 1단계에 비해서 29.7%가 유의하게 증가하였다. 따라서 가족가치관(β=.35, *p*<.001), 생식건강지식(β=.24, *p*<.001), 과거 생식기 질병 경험 중 생식기 감염(β=.09, *p*<.001)이 생식건강증진행위에 유의한 영향을 미치는 것으로 나타났다.

## Discussion

본 연구는 기혼여성을 대상으로 가족가치관, 생식건강지식을 확인하고 생식건강증진행위에 미치는 영향요인을 파악함으로써, 기혼여성들의 생식건강 및 생식건강증진행위를 돕는 간호 중재 프로그램 개발을 위한 기초자료를 제공하고자 시도되었다. 본 연구의 결과를 중심으로 논의하면 다음과 같다.

본 연구 대상자의 생식건강증진행위는 5점 만점에 평균 3.18점으로 비교적 낮은 수준으로 나타났다. 동일한 측정도구를 사용한 Lee와 Lee [14]의 생식건강증진행위 연구에서는 난임 여성 평균 3.98점과 비교하여 낮은 점수를 보였다. 이는 난임을 진단받고 생식건강증진을 위하여 적극적이고 긍정적인 실천 행위를 이행 중이므로 비교하기 어려운 부분이 있었다. 그리고 기혼여성들은 임신과 출산 후 육아, 가사노동 및 경제활동 병행으로 개인의 생식건강증진에 관심을 가지고 실천하는 것이 어려운 환경이므로 기혼여성들에게 맞는 차별화된 중재방법이 필요하다. 이는 Rahman 등[22]의 기혼여성을 대상으로 한 연구 결과를 바탕으로 생식건강증진행위 중재 개발 시 미디어를 통한 교육방법을 적극 활용하여 기혼여성의 시간과 공간적 제약의 부담을 줄이고 정보 습득률을 높일 수 있도록 하여야 한다.

본 연구 대상자의 가족가치관은 7점 만점에 평균 4.40점으로 비교적 낮은 수준으로 나타났다. 세부 항목으로 부모됨가치는 평균 4.45점, 자녀가치는 평균 4.36점이었다. 동일한 측정도구를 사용한 Seo [20]의 부모됨가치 평균 5.37점, 자녀가치 평균 4.90점과 비교하여 모두 낮은 점수를 보였다. 이는 급격한 사회변화로 인한 기혼여성들의 사회 진출 및 경제활동의 꾸준한 증가, 교육수준 향상, 초혼 연령 증가, 출산 연령의 증가 등의 원인으로 결혼과 출산에 대한 당위적 가치가 약화[8]된 것의 영향을 받은 것으로 생각된다.

본 연구 대상자의 생식건강지식은 34점 만점에 평균 27.98점으로 비교적 높은 수준으로 나타났다. 동일한 측정도구를 사용한 국내 결혼 이주여성의 생식건강지식을 확인한 연구[13]의 평균 18.28점보다 생식건강지식이 높았다. 그러나 평균 연령 34.23세이며 베트남, 중국, 필리핀 등 다양한 나라에서 국내로 이민 온 기혼 이주여성들에 대해서는 언어 이해능력, 나라별 교육수준, 문화적 차이 등을 감안해야 하므로 본 연구 대상자들과 생식건강지식 정도를 객관적으로 비교하기 어려운 부분이 있다. Fooladi 등[23]의 연구에서는 가임 여성들의 생식건강지식 확인은 의도하지 않은 임신 발생률을 줄이고, 생식건강에 긍정적인 행위를 장려하기 위하여 중요하며, 이는 가족 계획에까지 영향을 주므로 지역사회 역할과 생식생활 계획에 중요한 부분이라고 하였다. 반면에, 지금까지 국내 선행연구에서는 성 지식을 확인한 연구가 대부분이었다. 성 지식은 성 기관의 구조와 기능, 성행위, 임신 및 출산의 신체적, 생리적 현상에 대한 지식 중심으로 이루어져 있으나, 생식건강지식은 성 지식과 함께 가족 계획, 인공 임신중절, 성병, 성 건강 등의 건강문제를 총망라하는 광의의 개념이다[1]. 이에 여성 개인의 생식건강이 질병이 없는 상태뿐만 아니라 육체적, 정신적, 사회적으로 안녕한 상태를 위한 권리[5]가 중시되는 사회가 된 만큼 생식건강지식을 확인하고 여성들의 생식건강을 위한 간호중재를 개발해야 한다.

본 연구에서는 기혼여성의 생식건강증진행위에 미치는 영향 요인을 확인하기에 앞서, 생식건강증진행위, 가족가치관 및 생식건강지식 간의 정적 상관관계가 있는 것을 확인하였다. 이는 기혼여성들의 생식건강증진행위에 결혼, 출산, 자녀, 양육 등의 사회적 영향을 받는 주관적 인식인 가족가치관과 생식건강 관리와 개인의 임신 및 출산, 가족 계획에 올바른 의사결정을 할 수 있도록 돕는 생식건강지식이 의미 있는 관계임을 알 수 있다. 그리고 위계적 회귀분석을 시행한 결과, 가족가치관, 생식건강지식, 과거 생식기 질병 경험 중 생식기 감염이 영향요인으로 확인되었다. 가족가치관의 인식과 행태의 부모됨가치와 자녀가치는 기혼여성들의 생식건강증진행위에 영향을 준다는 것을 확인하였다. Lim과 Seo [6]의 연구에서 가족가치관의 자녀관의 자녀 필요성, 부모에 대한 가치가 긍정적일수록 출산 의향이 높아진다고 하였다. 이는 기혼여성의 가족가치관이 생식건강증진행위에 긍정적 영향을 미친다는 것을 지지해 준다. 이에 본 연구자는 결혼, 출산, 임신에 중점적이던 전통적 가족가치관과 함께 현대 가족가치관의 초혼 연령 증가와 출산 연령의 증가를 고려한 연령별 여성 생식건강의 기능 및 구조의 변화, 난임 예방 교육 및 생식건강 관리 방법, 고위험 산모를 위한 생식건강증진행위 등 구체적으로 구성하여 개인에게 맞춤형 생식건강 교육 및 관리 프로그램이 필요하다고 생각한다.

생식건강지식이 높을수록 생식건강증진행위가 높으며, 생식건강증진행위에 영향을 준다고 나타났다. 본 연구 결과와 함께 Park과 Choi [13]의 연구에서도 생식건강 지식이 생식건강증진행위를 통계적으로 유의하게 증가시켰다. 그러나 2020년 20–39세 기혼여성 427명을 대상으로 한 생식건강지식을 확인한 결과[24] 응답자의 절반 이상 생식건강지식 점수가 낮은 것으로 나타났으며, 2021년 20–49세 기혼여성 159명을 대상으로 생식건강지식을 확인한 연구[25]에서는 종교 및 민간요법에 따른 치료 의존도가 높고 생식건강지식은 낮은 것으로 확인되었다. 반면에, 국내에서는 2021년 10월부터 입법 예고된 모자보건법 개정안에서 생식건강과 관련한 정확한 지식 정보와 서비스의 제공을 명시[26]하므로 생식건강지식의 중요성이 커지고 있음을 확인할 수 있다. 이에 국내외 여성들의 생식건강증진행위를 증진하기 위해서는 개인의 생식건강 문제에 대한 올바른 평가와 대처 전략을 선택할 수 있게 하기 위한 생식건강지식 교육이 선행되어야 함을 상기시켜 준다. 그리고 생식건강지식 교육은 수요자 중심으로, 각 나라의 사회·문화·종교적 배경을 고려할 것을 함께 제언한다. 또한 기혼여성의 과거 생식기 질병 경험이 생식건강증진행위에 영향을 미친다는 결과는 과거 질병 경험을 통해 건강행위 실천 인식이 변화되어 생식건강증진행위에 영향을 줄 수 있음을 확인하였다. 특히 많은 여성들이 생식기 감염으로 병원 방문 시 질병에 따른 증상 치료와 함께 생식건강증진행위 중재 교육을 병행한다면 생식기 감염 예방과 올바른 생식건강증진행위 실천에 도움이 될 것으로 생각된다. 또한 병원마다 다양한 교육자료보다는 지자체에서 모자보건을 위한 일관성 있는 생식기 질병 분류에 따른 생식건강증진행위 교육자료를 제공함으로 여성 생식건강에 대한 신뢰 있는 의료정보를 공유하는 것도 긍정적인 방안으로 생각된다. 그리고 기혼여성의 생식건강을 지속적으로 유지하기 위한 생식기 질병 예방, 대처, 예후 관리가 포함된 생식건강증진행위 자가간호 설명서 개발이 필요하다고 생각된다. 나아가 Lee와 Lee [14]의 연구에서 생식기 질환과 생식기 감염이 난임의 연관 인자로 확인되었으므로, 여성 생식기 질병 및 증상에 국가 및 지역사회의 관심이 필요하다 하겠다.

본 연구에서 대상자의 일반적 및 생식건강 관련 특성에 따른 생식건강증진행위에 대하여 살펴보면 교육수준, 결혼 기간, 피임 방법, 과거 생식기 질병 경험이 통계적으로 유의한 차이를 보였다. 이전의 난임 및 정상여성[14], 미혼여성[27]을 대상으로 한 생식건강증진행위 연구에서는 ‘교육수준’에 따른 차이가 없었으나 본 연구에서는 교육수준에 따른 차이가 있는 것으로 나타났다. 대학교 그룹과 고등학교 이하 그룹에서 유의한 차이가 난 것은 대학교 그룹에서는 생식건강증진행위와 관련된 생식건강 정보를 받을 수 있는 고등교육 환경에 노출될 확률이 높기 때문으로 생각된다. 이에 생식건강증진행위 중재 교육 전에는 교육수준을 확인하고 25세 이상, 49세 미만의 기혼여성들에게 수준별 생식건강 교육 방법을 개발하여 생식건강 정보 격차를 줄이는 데에 도움이 되어야 한다. 그리고 결혼 기간에서는 1년 이상, 2년 미만 그룹에서 생식건강증진행위가 가장 높게 나타났다. 이는 2020년 신혼부부 통계청[28] 자료의 ‘결혼 후 평균 1년 5개월 뒤 첫째 아이를 낳는다’는 내용을 근거로 기혼여성들은 2년 미만 내 건강한 자녀를 낳기 위하여 임신을 준비하고 유지하는 과정에서 생식건강에 대한 긍정적 행위를 적극적으로 실천하고 있음을 확인하였다. 이처럼 혼인 연령과 출산 연령이 올라가고 있는 사회‧문화적 변화에서 기혼여성들의 연령에 따른 신체구성 및 체력을 반영한 생식건강증진행위 연구가 진행되어야 한다. 또한 기혼여성의 피임 방법에 따라 생식건강증진행위에 유의한 차이를 보였다. 피임 방법에서 콘돔이 가장 높게 나타난 것은 인체에 부작용이 거의 없고, 사용이 간편하며 구입이 쉽기 때문이다. 그리고 피임 확률이 낮은 월경주기법과 ‘피임을 하지 않는다’는 항목에 표시한 대상자들도 있었다. 이를 바탕으로 학생, 미혼여성 대상의 다양한 피임 교육을 기혼여성을 대상으로 확대해야 한다. 기혼여성들은 인공 임신중절 경험률이 높고, 생식기 건강문제를 가지고 있는 빈도가 높으므로[12], 생식건강 증진행위 교육 시 피임의 필요성과 방법에 따른 장·단점을 필수적으로 포함시켜야 함을 확인하였다. 과거 생식기 질병 경험에 따른 생식건강증진행위 차이를 살펴보면, 생식기 감염 그룹에서 양성종양 질병 그룹과 월경 장애 그룹에 비해 높게 나타났다. 대부분 양성종양 질병은 진단과 함께 대증치료 및 수술로 인하여 생식건강증진행위에 대한 관심과 실천이 시작되고, 월경장애는 호르몬 변화에 따라 나타나는 증상으로 지속적인 생식건강증진행위보다는 일시적인 증상 완화 대처가 우선되는 반면, 생식기 감염 대상자들은 성생활, 면역, 스트레스 등의 요인으로 발생되며 가려움, 분비물, 통증 등의 증상으로 생식기 관련 개인위생 관리 실천율이 높은 것으로 생각된다. 또한 Lee [19]의 연구에서도 생식기 감염 질환 경험이 있는 경우 생식건강증진행위의 수행이 높다고 나타났다.

본 연구는 대상자 수집 과정에서 직장 여성의 비율이 높고, 월평균 수입을 구체적으로 확인하지 못한 한계점이 있었다. 그러나 기혼여성을 대상으로 가족가치관과 생식건강지식이 생식건강증진행위에 미치는 영향을 확인함으로써 생식건강증진행위가 성행위에 대한 책임감, 생식건강 관리, 임신 및 출산과 관련한 가족 계획을 내포하여 기혼여성의 안녕에 본질적으로 중요하다[29]는 것을 규명하였다.

## Figures and Tables

**Table 1.** Differences in RHPB according to the characteristics of participants (N=170)

Characteristics	Categories	n (%)	Categories (mean±SD)	RHPB (mean±SD)	t or F	*p*	Scheffé
*General characteristics*							
Age (year)	<30	29 (17.1)	32.28±0.31	3.53±0.20	0.36	.522	
	30-34	75 (44.1)		3.48±0.25			
	35-39	36 (21.2)		3.52±0.11			
	40-44	22 (12.9)		3.11±0.11			
	≥45	8 (4.7)		3.02±0.41			
Religion	Yes	132 (77.6)		3.52±0.18	0.23	.318	
	No	38 (22.4)		3.55±0.31			
Level of education	≤ High school	2 (1.1)		3.27±0.22	1.32	.011	a<c
	Junior college	27 (15.9)		3.30±0.17			
	University	123 (72.4)		3.51±0.38			
	≥Graduate School	18 (10.6)		3.32±0.41			
Job	Yes	158 (92.9)		3.70±0.11	2.65	.111	
	No	12 (7.1)		3.18±0.28			
Income per month (×10^4^ KRW)	<150^a^	11 (6.5)	251.09±0.58	3.24±0.16	1.34	.410	
	150-299^b^	108 (63.5)		3.60±0.21			
	300-449^c^	35 (20.6)		3.32±0.18			
	≥450^d^	16 (9.4)		3.35±0.11			
*Reproductive health characteristics*							
Duration of marriage (year)	<1^a^	24 (14.1)	2.62±0.92	3.50±0.15	0.33	.011	b>c
	1 – 2^b^	56 (32.9)		3.65±0.18			
	3 – 4^c^	51 (30.0)		3.40±0.11			
	≥5^d^	39 (22.9)		3.49±0.28			
Children	Yes	122 (71.8)		3.56±0.23	3.74	.333	
	No	48 (28.2)		3.39±0.11			
Reproductive health status	Best	13 (7.6)		3.43±0.44	1.41	.288	
	Good	34 (20.0)		3.49±0.20			
	General	101 (59.4)		3.51±0.88			
	Poor	17 (10.0)		3.65±0.08			
	Worst	5 (2.9)		3.44±0.22			
Contraceptive method	Condom^a^	92 (54.1)		3.56±0.18	1.01	.021	a>d
	Oral	26 (15.3)		3.48±0.25			
	Contraceptive pill^b^						
	Coitus interruptus^c^	19 (11.2)		3.48±0.22			
	Natural family planning^ d^	17 (10.0)		3.44±0.16			
	Intrauterine device^e^	4 (2.3)		3.49±0.30			
	None^f^	12 (7.1)		3.40±0.34			
Experience of reproductive diseases	Menstrual disorder^a^	68 (40.0)		3.49±0.32	0.55	.018	a<b
	Genital infection^b^	47 (27.6)		3.67±0.17			
	Benign tumor^c^	55 (32.4)		3.58±0.18			
Ever received RH education	Yes	67 (39.4)		3.47±0.34	2.72	.147	
	No	103 (59.2)		3.53±0.55			
Perceived necessity of RH education	Yes	149 (87.6)		3.62±0.52	2.55	.393	
	No	21 (12.4)		3.48±0.21			

RHPB: Reproductive health-promoting behaviors; KRW, Korean won (1 million KRW is roughly 800 US dollars); RH: reproductive health.

**Table 2.** RHPB, family values, and reproductive health knowledge (N=170)

Variable	Possible range	Minimum	Maximum	Mean±SD
RHPB	1–5	2.26	5.00	3.18±0.18
Safe sex behavior	1–5	2.67	5.00	3.32±0.17
Sexual responsibility	1–5	2.00	5.00	3.10±0.35
Genital health management	1–5	2.00	5.00	3.09±0.23
STI prevention	1–5	2.65	5.00	3.31±0.21
Genital hygiene management	1–5	2.00	5.00	3.09±0.25
Family values	1–7	1.37	7.00	4.40±0.57
Value of being a child	1–7	1.00	7.00	4.36±1.02
Value of being a parent	1–7	1.75	7.00	4.45±1.01
Reproductive health knowledge	0–34	18.00	34.00	27.98±0.25
Structure and function of reproductive system	0–6	1.18	6.00	4.44±0.52
Pregnancy and childbirth	0–11	1.00	11.00	10.13±0.18
Contraception and STI	0–12	1.00	12.00	8.55±0.09
Cancer of the reproductive system	0–5	1.32	5.00	3.88±0.71

RHPB: Reproductive health-promoting behaviors; STI: sexually transmitted infection.

**Table 3.** Relationships among RHPB, family values, and reproductive health knowledge (N=170)

Variable	r (*p*)		
	RHPB	Family values	RH Knowledge
RHPB	1		
Family values	.78 (<.001)	1	
RH knowledge	.55 (<.001)	.41 (.018)	1

RH: reproductive health; RHPB: Reproductive health-promoting behaviors.

**Table 4.** Factors affecting reproductive health-promoting behaviors (N=170)

Variable	Model 1				Model 2			
	B	β	t	*p*	B	β	t	*p*
Level of education								
≤High school	–0.20	–.27	–0.19	.321	−0.32	–.27	−0.44	.302
Junior college	–0.05	–.23	–0.45	.142	–0.25	–.10	–0.54	.071
≥Graduate School	–1.04	–.21	–0.86	.074	–0.21	–.41	–0.27	.198
Duration of marriage (years)								
<1	0.65	.41	0.68	.061	0.18	.44	1.74	.120
3–4	0.08	.18	0.54	.101	0.21	.41	0.84	.186
≥5	0.38	.25	1.89	.301	0.12	.38	0.35	.052
Contraceptive method								
Oral contraceptive pill	0.19	.58	1.67	.807	0.14	.24	0.28	.074
Coitus interruptus	0.44	.09	0.72	.340	1.21	.41	2.41	.094
Menstrual cycle	0.67	.14	1.78	.257	0.54	.13	1.18	.057
Intrauterine device	1.98	.23	2.67	.080	0.27	.13	1.87	.082
Experience of reproductive diseases								
Genital infection	0.93	.13	1.62	<.001	0.86	.09	1.24	<.001
Benign tumor	0.64	.12	1.58	.720	0.39	.21	0.64	.252
Family value					0.86	.35	0.87	<.001
Reproductive health knowledge					1.12	.24	1.47	<.001
R^2^	.101				.415			
Adjusted R^2^	.215				.512			
F (*p*)	18.48 (<.001)				41.07 (<.001)			

Reference groups were level of education (university), duration of marriage (1-2 years), contraception method (condom), and experience of reproductive diseases (menstrual disorder).
